# Association between Statin Use and Chemotherapy-Induced Cardiotoxicity: A Meta-Analysis

**DOI:** 10.3390/medicina60040580

**Published:** 2024-03-31

**Authors:** Vikash Jaiswal, Song Peng Ang, Novonil Deb, Muhammad Hanif, Nitya Batra, Sai Gautham Kanagala, Nikhil Vojjala, Kripa Rajak, Poulami Roy, Medha Sharath, Madeeha Subhan Waleed, Zarghoona Wajid, Jishanth Mattumpuram

**Affiliations:** 1 Department of Cardiovascular Research, Larkin Community Hospital, South Miami, FL 33143, USA; 2 Department of Internal Medicine, Rutgers Health/Community Medical Center, Toms River, NJ 08755, USA; 3 North Bengal Medical College and Hospital, Darjeeling 734012, West Bengal, India; 4 Department of Internal Medicine, SUNY Upstate Medical University, 750 E Adams St., Syracuse, NY 13210, USA; 5 Department of Internal Medicine, Beaumont Hospital, Royal Oak, MI 48073, USA; 6 Department of Internal Medicine, Metropolitan Hospital Center, New York, NY 10029, USA; 7 Internal Medicine Department, Trinity Health Oakland/Wayne State University, Detroit, MI 48341, USA; 8 Department of Internal Medicine, UPMC Harrisburgh, 111 S Front St., Harrisburg, PA 17101, USA; 9 Bangalore Medical College and Research Institute, Kalasipalya, Bengaluru 560002, Karnataka, India; 10 Department of Internal Medicine, Lower Bucks Hospital, Bristo, 501 Bath Rd., Bristol, PA 19007, USA; 11 Department of Internal Medicine, Wayne State University School of Medicine, 540 E. Canfield Ave., Detroit, MI 48201, USA; 12 Division of Cardiology, Department of Medicine, University of Louisville School of Medicine, Louisville, KY 40202, USA

**Keywords:** statins, chemotherapy, cardio-oncology, heart failure

## Abstract

*Background:* Chemotherapy-induced cardiac dysfunction (CIC) is a significant and concerning complication observed among cancer patients. Despite the demonstrated cardioprotective benefits of statins in various cardiovascular diseases, their effectiveness in mitigating CIC remains uncertain. *Objective:* This meta-analysis aims to comprehensively evaluate the potential cardioprotective role of statins in patients with CIC. *Methods:* A systematic literature search was conducted using PubMed, Embase, and Scopus databases to identify relevant articles published from inception until 10th May 2023. The outcomes were assessed using pooled odds ratio (OR) for categorical data and mean difference (MD) for continuous data, with corresponding 95% confidence intervals (95% CIs). *Results:* This meta-analysis comprised nine studies involving a total of 5532 patients, with 1904 in the statin group and 3628 in the non-statin group. The pooled analysis of primary outcome shows that patients who did not receive statin suffer a greater decline in the LVEF after chemotherapy compared to those who receive statin (MD, 3.55 (95% CI: 1.04–6.05), *p* = 0.01). Likewise, we observed a significantly higher final mean LVEF among chemotherapy patients with statin compared to the non-statin group of patients (MD, 2.08 (95% CI: 0.86–3.30), *p* > 0.001). Additionally, there was a lower risk of incident heart failure in the statin group compared to the non-statin group of patients (OR, 0.41 (95% CI: 0.27–0.62), *p* < 0.001). Lastly, the change in the mean difference for LVEDV was not statistically significant between the statin and non-statin groups (MD, 1.55 (95% CI: −5.22–8.33), *p* = 0.65). *Conclusion:* Among patients of CIC, statin use has shown cardioprotective benefits by improving left ventricular function and reducing the risk of heart failure.

## 1. Introduction 

Cancer is one of the leading causes of morbidity and mortality worldwide, accounting for one in every six deaths, second only to cardiovascular diseases. The incidence of cancer was estimated at 18.1 million cases causing 9.9 million cancer deaths globally in 2020 [1]. Cancer treatment has successfully reduced the cancer load by a significant proportion through therapeutic advances and lowering the risk of recurrence. However, this wonder treatment often carries a significant hurdle of application due to adverse events [2]. Chemotherapy is one of the principal modalities of management and essentially exerts toxic effects on the fast-growing neoplastic cells. It is associated with a plethora of short- and long-term side effects due to collateral damage endured by normal cells. One of such important side effects is CIC [3]. Cardiotoxicity is a well-pronounced adverse effect associated with chemotherapy which directly affects the clinical outcomes [3]. Although a clear definition and clinical criteria regarding the diagnosis of CIC was lacking, an expert committee supervising the trastuzumab clinical trials finally established a few landmarks; the presence of one or more could be defined as CIC [4]: (1) a reduction in the LVEF, globally or specific to the interventricular system, (2) signs and symptoms promoting towards heart failure, and (3) a reduction in the LVEF from baseline ≤ to 5% to <55% in the presence of signs or symptoms of heart failure (HF) or a reduction in the LVEF ≥10% to <55% without signs or symptoms of HF [4]. CIC can be acute, subacute, or chronic. Acute events may develop anytime in the timespan between the initiation of the treatment up to two weeks after treatment completion. They are characterized by events like arrhythmias, QT abnormalities, ventricular repolarization, acute coronary syndrome, and myocardial dysfunction [5]. On the other hand, chronic events generally range from one year after the completion of treatment to more. They are mostly asymptomatic and have underlying systolic and diastolic dysfunction which may result in irreversible cardiac failure or even death [6]. Drugs most notorious for causing CIC belong to the anthracycline group, taxanes and anti-HER2 receptor antibodies, widely used in breast cancers and lymphomas [7]. A recent study by Cardinale et al. showed that anthracycline-induced cardiotoxicity was associated with 45% of patients having no improvement in the LVEF even after catered treatment, while trastuzumab showed reversible cardiotoxicity after the stoppage of treatment; also, after starting neurohormonal antagonists for heart failure, many patients were able to handle another challenge of trastuzumab [5,8,9]. Therefore, the assessment of CIC prior to starting chemotherapeutic drugs is essential for targeted treatments and better clinical outcomes. The cardiotoxic effects are multifactorial due to the (1) production of reactive oxygen species, mitochondrial dysfunction, and cytokine release, which promotes myocyte injury and results in systolic dysfunction, (2) replacement fibrosis, (3) progress to irreversible heart failure, (4) coagulation dysfunction resulting in thrombogenesis and vascular injury, and (5) myocardial or pericardial inflammation [10]. The estimated incidence of CIAC is variable in different studies due to the usage of multiple classification systems; it has been estimated to be <1% with taxanes, 2–20% with trastuzumab, and as high as up to 48% with doxorubicin [11]. 

Statins have shown great promise in preventing CIC [11,12]. It is now well known that statins exert their cardioprotective effects not only through the direct lipid-lowering mechanism by inhibiting the rate-limiting enzyme HMG COA 3-hydroxy-3- methylglutaryl-coenzyme A (HMG-CoA) reductase but also a multitude of pleiotropic anti-inflammatory effects like the enhanced expression of antioxidative enzymes and decreased production of proinflammatory cytokines such as tumor necrosis factor-alpha (TNFα) [13]. Current practice is to commence cardiac treatment only after cardiomyopathy is overt and often irreversible. As CIC is often subclinical initially and progresses to heart failure, this approach leads to incomplete recovery of heart function and increases the risk of adverse cardiovascular events [14]. 

Multiple animal model studies, observational studies, and small-scale prospective studies about preemptive statin use to prevent CIAC have shown promising results [11,12,15]. On the other hand, the PREVENT trial showed no advantage of statins in preventing LVEF reduction [16]. Hence, the current picture is mixed with inconsistent results. The main objective of this study is to gather and comprehensively review the existing literature about the preemptive use of statin in mitigating CIAC in order to address one of the major setbacks in chemotherapy patients. We sought to perform an updated meta-analysis, including the most recently published randomized controlled trials such as PREVENT, STOP CA, and SPARE-HF, to evaluate the association between statin use and the incidence of heart failure and the change in the LVEF [16,17,18].

## 2. Methods

This meta-analysis was conducted and reported following the PRISMA (preferred reporting items for systematic review and meta-analysis) 2020 guidelines and performed according to established methods, as described previously [19,20,21,22].

### 2.1. Search Strategy

We conducted a systematic literature search in PubMed, Embase, and ClinicalTrial.gov using predefined MESH terms by using “AND” and “OR”. The following search terms were used: “statin” AND “chemotherapy” OR “anthracyclines” AND “breast cancer” OR “lymphoma” AND “neoplasm” AND “cardiomyopathy” OR “heart failure” OR “ischemic heart disease”. We queried databases from their search inception up until 15th April 2023 without any restrictions on the language of the studies. Search strategies are listed in Supplementary Table S1. 

All the studies were carefully screened and exported to the Mendeley reference manager used to handle searched citations. A manual check was carried out to crosscheck for any remaining duplicates. Two reviewers (ND and MH) reviewed the papers based on the title and abstract. Discrepancies regarding the inclusion of studies were arbitrated by another author (MSW).

### 2.2. Eligibility Criteria 

We included studies with adult patients ≥18 years of age. All randomized controlled trials and prospective and retrospective studies were considered to be eligible if the included patients had cancer and were on chemotherapy. It was decided to include studies with two arms with statin users as an intervention group and non-statin users as a control group. Studies were required to report outcomes of interest such as change in mean LVEF among statin users and in non-statin users, mean final LVEF, change in mean left ventricular end-diastolic volume, and heart failure incidence. Selected studies compared patients with varying baseline characteristics and comorbidity. 

Studies that were performed on animals, reviews, case reports, case series, studies on patients <18 years, studies with a single arm or non-cancer patients, and studies without statin as an intervention or without outcomes of interest were excluded from this review.

## 3. Clinical Outcomes

The primary outcome of this meta-analysis was a change in the mean LVEF among statin users and in non-statin users. Secondary outcomes include the mean final LVEF, change in mean left ventricular end-diastolic volume, and heart failure incidence.

## 4. Data Extraction and Quality Assessment

Data from the eligible studies, such as demographics, study design, comorbidity, and outcomes between statin and non-statin groups, were extracted to a Microsoft Excel^®^ 2019 spreadsheet by two authors (ND and PR). 

Two authors (V.J. and VA) independently assessed the quality of the included studies using the Newcastle Ottawa scale for observational studies and Cochrane Collaboration’s tool for assessing the risk of bias in randomized controlled trials [23,24].

## 5. Statistical Analysis

Baseline continuous variables were summarized as a mean (standard deviation), whereas dichotomous variables were described as a frequency or percentage. We performed a conventional meta-analysis for primary and secondary outcomes and adopted the DerSimonian and Laird random-effect model for the study variations [25]. Outcomes were reported as mean difference (MD) for continuous data and pooled odds ratio (OR) for categorical data with their corresponding 95% confidence intervals (CIs). Statistical significance was met if the 95% CI did not cross the numeric “1” and the two-tailed *p*-value was less than 0.05. We considered a two-tailed *p*-value of less than 0.05 to be statistically significant. In addition, we assessed the between-study heterogeneity using the Higgins I-square (I^2^) test, with I^2^ values < 75% considered mild–moderate and >75% considered high [26]. All statistical work, inclusive analysis, and graphical illustrations were conducted using STATA (version 17.0, StataCorp) [27]. 

## 6. Results

### 6.1. Baseline Characteristics of Patients in Included Studies

**Figure 1:**
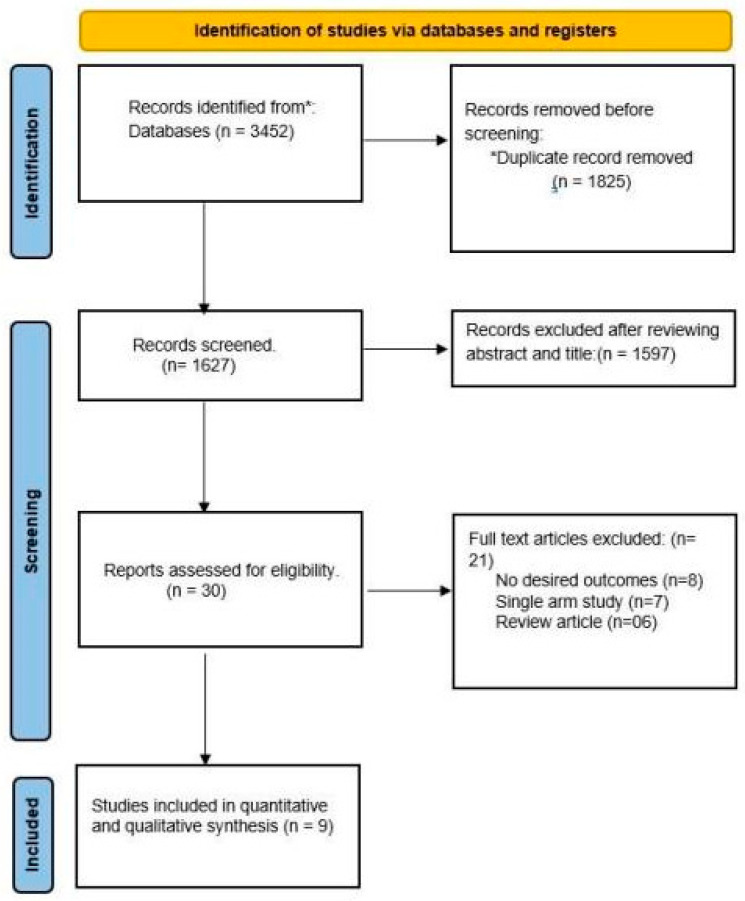
PRISMA flow diagram for search strategy.

The initial search strategy yielded 3452 articles, from which 1825 duplicates were removed, and 1597 articles were excluded after the title and abstract screening. A full-text review was performed on the remaining 30 studies, after which 21 studies were excluded from the final review and analysis for the following reasons: lack of appropriate comparison arm, wrong population, non-statin group, or lack of outcome of interest (Figure 1).

In summary, nine studies met the eligibility criteria and were included in the final analysis [11,12,15,16,17,18,28,29,30]. Five studies were randomized controlled trials [12,16,17,18,28] and four were observational cohort studies [11,15,29,30]. STOP-CA was a double-blind randomized trial that involved 300 patients with lymphoma who were on anthracycline [16]. Patients were randomized with 40 mg/d atorvastatin orally in the intervention group. Another randomized trial by Nabati et al. included randomized breast cancer patients with rosuvastatin 20 mg for preventing cardiotoxicity [18]. Another RCT by Hundley et al. and SPARE-HF enrolled breast cancer and lymphoma patients, with the intervention group on atorvastatin therapy [17,28]. Another study by Acar et al. included randomized cancer patients with atorvastatin as an intervention [12]. The total number of patients was 5532, with 1904 patients in the statin group and 3628 in the non-statin group. The mean age of patients among the statin and non-statin groups was 58.12 and 55.54 years. The most common comorbidities among both groups of patients include hypertension (75.8% vs. 24.2%) and diabetes mellitus (64.8% vs. 35.2%) among statin and non-statin groups of patients. The study characteristics, patients’ demographics, and comorbidities are presented in Table 1. 

### 6.2. Meta-Analysis of the Outcomes among Cancer Patients with Statin and Non-Statin Users

**Figure 2:**
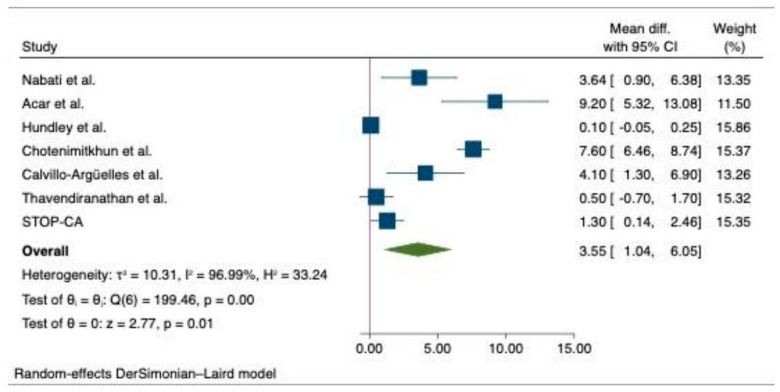
Forest plots of primary outcome—difference in the decline in LVEF between statin and non-statin users [12,15,17,18,28,29].

**Figure 3:**
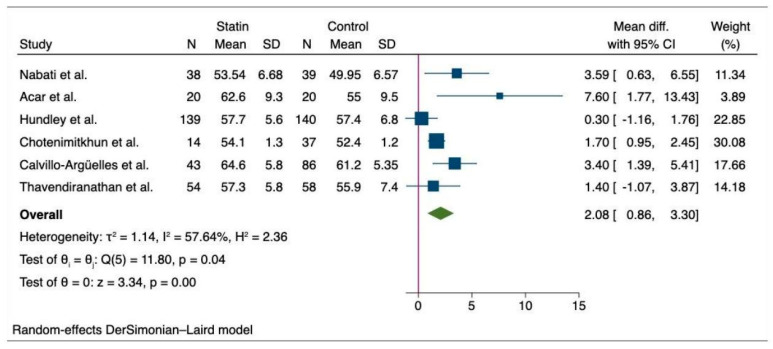
Forest plot of secondary outcome—difference in the final mean LVEF between statin and non-statin users [12,15,17,18,28,29].

**Figure 4:**
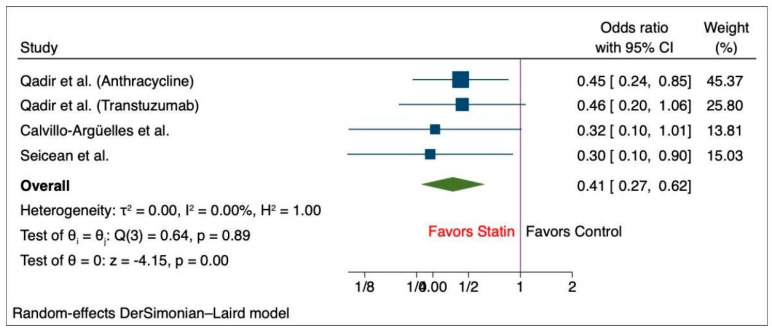
Forest plot of secondary outcome—incidence of heart failure [11,15,30].

**Figure 5:**
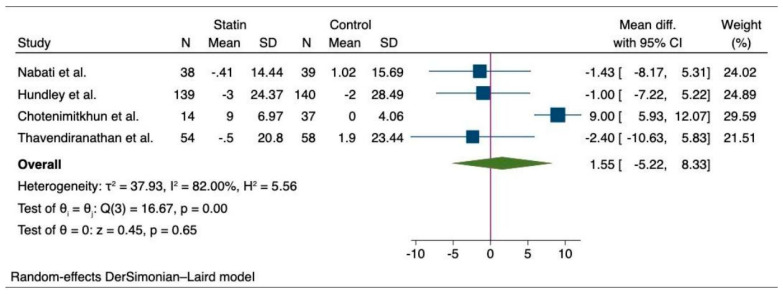
Forest plot of secondary outcome—change in pre- and post-treatment LVEDV between statin and non-statin users [17,18,28,29].

The pooled analysis of primary outcome shows that patients who did not receive statin suffered a greater decline in the LVEF after chemotherapy compared to those who received statin (MD, 3.55 (95% CI: 1.04–6.05), *p* = 0.01, I^2^ = 96.99%) (Figure 2). Likewise, we observed a significantly higher final mean LVEF among chemotherapy patients with statin compared to the non-statin group of patients (MD, 2.08 (95% CI: 0.86–3.30), *p* > 0.001, I^2^ = 57.64%) (Figure 3). Additionally, there was a lower risk of incident heart failure in the statin group compared to the non-statin group of patients (OR, 0.41 (95% CI: 0.27–0.62), *p* < 0.001), I^2^ = 0%) (Figure 4). Lastly, the change in pre- and post-treatment LVEDV was not statistically significant between the statin and non-statin groups (MD, 1.55 (95% CI: −5.22–8.33), *p* = 0.65, I^2^ = 82%) (Figure 5).

## 7. Quality Assessment, Sensitivity Analysis, and Publication Bias

The quality assessment using NOS for observational studies and Cochrane risk of bias for randomized trials showed a low risk of bias across studies (Supplementary Table S2 and Figure S1). We further conducted a sensitivity analysis on outcomes with high heterogeneity (>75%), which are the change in the LVEF and change in LVEDV. After conducting a leave-one-out analysis, these results remained largely consistent with the primary analysis, which is that the decline in the LVEF remained significantly greater in non-statin users compared to statin users while the change in LVEDV remained non-significant between both groups of patients (Supplementary Figures S4 and S5). The assessment of publication bias was conducted through a funnel plot. Through the visualization of the funnel plot, there was a funnel plot asymmetry observed for the change in the LVEF, indicating the possibility of publication bias (Supplementary Figure S2). The funnel plot appears symmetrical for heart failure (Supplementary Figure S3). 

## 8. Discussion

To the best of our knowledge, this is the most comprehensive meta-analysis with the highest sample size thus far, highlighting statin’s role in preventing chemotherapy-induced cardiovascular dysfunction. In this study, we revealed that while there was a decrease in the LVEF in both groups after chemotherapy, the patients who did not receive statin experienced a statistically significant greater decline in the LVEF as compared with the statin-taking cohort. Similarly, we found a significantly higher final mean LVEF in statin-treated cohorts as compared with non-statin cohorts in chemotherapy patients. Additionally, the statin group was found to have a significantly reduced risk of heart failure incidence as compared with the control group. However, we did not find any significant difference in left ventricular end-diastolic volume in both the cohorts, i.e., statin-taking and non-statin cohort.

Nabati et al. conducted a study to evaluate the effect of statin in women with breast cancer receiving chemotherapy and revealed that in the placebo group there was a significant LVEF reduction in comparison to the statin-treated group, findings concordant with our study [18]. Similarly, Calvillo-Argüelles et al. conducted a study to evaluate statin’s cardioprotective effect in patients receiving anti-HER2 therapy, and they reported that the LVEF was found to be significantly lower in the placebo group compared to those receiving statin, which further supports our results [15]. However, Hundley WG et al. did not reveal any significant difference in the LVEF decline after two years of doxorubicin therapy in the statin-treated cohort as compared to the control cohort, results discordant with our findings [28]. Statins were also found to be beneficial in decreasing the incidence of heart failure after receiving chemotherapy. A propensity-matched cohort study reported that the five-year heart failure incidence after anthracyclines was significantly reduced in statin-treated women compared to unexposed women (3.7%), supporting our results [11]. Similarly, in another observational study, significant reductions were found in the incidence of heart failure in statin-treated groups after receiving anthracyclines chemotherapy as compared to the control group [30].

Although many chemotherapeutic drugs have potential cardiotoxicity, the most commonly reported are anthracyclines, fluoropyrimidines, taxanes, alkylating agents, and monoclonal antibodies. The most commonly used, anthracycline and doxorubicin, used in leukemia, lymphoma, and other solid tumors like breast, ovary, lung, and stomach, are tolerated well up to a cumulative dose of 300 mg/m^2^, with a heart failure rate of less than 2% [31]. The majority of cardiotoxicity secondary to anthracyclines has been reported within the first year of therapy (up to 98%); however, rarely, it has been reported after many years [32]. Fluoropyrimidines are the second most common class of cardiotoxic chemotherapeutic drugs reported, generally well tolerated; nevertheless, 1–18% of patients experience cardiotoxicity in the form of reversible coronary artery spasm and myocardial ischemia as well as directly through cardiomyocyte-intrinsic mechanisms [33]. Taxanes, such as paclitaxel, have been reported to induce cardiotoxic events in 3–20% of patients and include QT prolongations followed by bradycardia and atrial fibrillation [34]. Cardiotoxicity due to alkylating agents such as cisplatin and cyclophosphamide has also been reported in the literature. Cisplatin-based chemotherapy has been reported to cause cardiovascular diseases such as angina and myocardial infarction in 7–32% of patients [35]. Long-term unfavorable cardiovascular risks such as hypercholesterolemia, hypertriglyceridemia, hypertension, and insulin resistance have been reported in cisplatin-treated patients. Other cardiotoxic chemotherapeutic agents reported are monoclonal antibodies like trastuzumab and pertuzumab, and less commonly, tyrosine kinase inhibitors such as imatinib have been reported in the literature. Experts including cardiologists and oncologists recommend identifying patients with pre-existing coronary artery disease before starting cancer treatment and performing a cardiac ultrasound and repeating it one year after treatment with cardiotoxic chemotherapeutic agents, i.e., anthracyclines [a]. They also recommend emphasizing the importance of creating multidisciplinary teams including oncologists, cardiologists, surgeons, radiotherapists, gastroenterologists, and endocrinologists at every step of cancer treatment [a]. 

Statins are considered among the first-line pharmacotherapy for individuals with increased cardiovascular risk [36]. The primary use of statins is to lower cholesterol, particularly low-density lipoprotein (LDL) via the inhibition of 3-Hydroxy-3-methylglutaryl coenzyme-A reductase (HMG-CoA reductase). However, all the beneficial effects of statin in reducing cardiovascular risks cannot be explained solely by reductions in LDL. Statins are also found to inhibit Rho-kinases, independent of changes in cholesterol level, and Rho-kinases are considered as a pro-atherogenic enzyme [37]. The inhibition of Rho-kinases contributes to several cardiovascular protective effects, in part through the reduction in the NFkB signaling pathway, which subsequently leads to the enhanced activity of endothelial nitric oxide synthase and increased nitric oxide availability, which stimulates troponin-1 phosphorylation and myocardial relaxation [38]. This suggests that statin plays a vital role in long-term cardiac remodeling through nitric oxide-induced mechanisms. Additionally, increases in nitric oxide have been linked to a decrease in vascular inflammation and endothelial dysfunction [39]. Massaro M et al. conducted a study and revealed that statins have protective effects against plaque angiogenesis and rupture via matrix metalloproteinase-9 (MMP-9) and cyclooxygenase-2 (COX-2) pathways [40]. Additionally, statins have been found to improve vascular barrier function and reduce the permeability of inflammatory leukocytes into the infarcted tissue, consequently improving left ventricular outcomes [41]. Thus, these direct and indirect actions of statins reduce overall cardiovascular risk and atherosclerosis.

In addition to the cardioprotective effects of statins, the previous literature has shown that statins can also play a vital role in reducing the overall incidence of cancer and mortality associated with it. Graaf MR et al. conducted a study to evaluate the effect of statin on cancer risk reduction and revealed that statin is associated with a 20% risk reduction in cancer as compared with the control [42]. Ren WQ conducted a study to evaluate the role of statin in cancer risk reduction and mortality associated with it in heart failure patients and found that statin-treated cohorts were associated with a significant reduction in cancer incidence as compared to non-users [43]. Similarly, mortality associated with cancer was also significantly lower in statin users as compared to non-users [43]. Another study conducted by Chang WT et al. on the Asian population revealed that statin users have lesser cancer-related mortality as compared to non-users [44]. The exact mechanism through which statin reduces cancer mortality and cancer incidence is still debatable. A study demonstrated that statin decreases the cholesterol contents of lipid rafts in malignant prostatic cells, which hinders AKT signaling and induces apoptosis [45]. Additionally, statin also reduces the production of geranylgeranyl pyrophosphate (GGPP), a non-sterol isoprenoid, and certain cancer cells rely on GGPP for survival [46]. In one study, statins were studied against seven different types of solid tumors, including breast, ovarian, prostate, colon, lung, brain cancer, and melanoma, and it was revealed that statins affect the proliferation of each tumor differently [47]. The study revealed that the growth of some cell lines was fully suppressed and some was partially suppressed, while some cell lines were insensitive to statin treatment, suggesting that the pharmacological effect is mainly influenced by the genetic background of cancer cells [47,48]. 

Hydrophilic statins such as rosuvastatin and pravastatin, which mainly inhibit cholesterol biosynthesis in the liver, are less bioavailable in the peripheral tissues as compared with lipophilic statins such as atorvastatin and simvastatin, and thus lipophilic statins provide potential mevalonate metabolism in cancer cells [49]. Cardwell CR et al. conducted a study to evaluate the survival benefits of statins in newly diagnosed lung cancer patients [50]. They revealed that statin users, especially those using lipophilic statins, i.e., simvastatin, were associated with a reduced rate of cancer-related mortality, showing that lipophilic statins had better survival benefits than hydrophilic statins in cancer [50]. Another study was conducted by Chou CW et al. to investigate the effect of statin on lung cancer and reported that patients with p53 mutations had a decreased 5-year mortality in a simvastatin-treated cohort [51]. They concluded that simvastatin has a high tumor-suppressive effect in both mutant and normal p53 cancer cells by regulating different cell pathways [51]. Thus, the literature shows that among statins, lipophilic statins, such as atorvastatin and simvastatin, have more tumor-suppressive effects as compared with hydrophilic statins like rosuvastatin.

Various pharmacological and non-pharmacological strategies have been applied in the past to prevent chemotherapy-induced cardiotoxicity and have been successful. In addition, lowering cholesterol, controlling blood pressure, maintaining healthy blood glucose, smoking cessation, consuming a healthy diet, and moderate aerobic exercise are techniques that can further reduce chemotherapy-induced cardiotoxicity [52]. The only FDA-approved cardioprotective agent for anthracycline-induced cardiotoxicity is dexrazoxane, and this drug changes the configuration of the enzyme topoisomerase II and prevents its binding with doxorubicin [53]. Studies have proven the beneficial effect of dexrazoxane in the reduction in clinical heart failure and cardiac events in patients receiving anthracycline chemotherapy [54]. However, dexrazoxane is only FDA-approved for those breast cancer patients who have already received >300 mg/m^2^ of the doxorubicin therapy, and this threshold could be because it interferes with doxorubicin antitumor efficacy [55].

The literature shows that in addition to statins, angiotensin-converting enzyme inhibitors (ACEIs), B-blockers, and aldosterone antagonists also have a favorable effect on the LVEF in chemotherapy-treated patients. A meta-analysis conducted by Dong H et al. evaluated the effects of ACEIs/ARBs in preventing cardiotoxicity caused by chemotherapy in early-stage breast cancer and revealed that ACEI-/ARB-treated groups have a significantly higher LVEF as compared to the control group [56]. However, they also found that the incidence of cardiac events in ACEIs/ARBs was not significantly lower than in the control group [56]. Another study conducted by Lin H et al. to evaluate the cardioprotective effects of ACEIs/ARBs in anthracycline-induced chronic cardiotoxicity reported that the prophylactic use of ACEIs/ARBs has potential benefits for anthracycline-induced cardiotoxicity [57]. They found that the left ventricular ejection fraction (LVEF) was better preserved in the ACEI-/ARB-treated cohort as compared with controls, showing the beneficial effect of ACEIs/ARBs in preventing chemotherapy-induced cardiotoxicity [57]. Similarly, Cardinale D et al. found that early treatment with ACEIs seems to prevent late cardiotoxicity from chemotherapy [58]. The effects of beta-blockers for the primary prevention of anthracycline-induced cardiotoxicity were studied by Ma Y et al., who found that beta-blockers were associated with a significant improvement in the LVEF as compared to the placebo [59]. Pituskin E et al. conducted a trial to evaluate the protective effect of bisoprolol in patients with breast cancer receiving trastuzumab. They demonstrated a decline in the LVEF attenuated in the bisoprolol-treated cohorts as compared to the placebo [60]. Another trial conducted by Avila MS et al. to evaluate the effect of carvedilol on the prevention of chemotherapy-related toxicity revealed a significantly decreased incidence of cardiac diastolic dysfunction and troponins level in the carvedilol-treated cohort as compared with the placebo; however, no significant differences were noted in the LVEF in both cohorts [61]. Similarly, aldosterone antagonists were evaluated in breast cancer patients receiving anthracycline chemotherapy, and it was reported that spironolactone administration protects myocardial functions [62]. Thus, the literature shows that in addition to statins, beta-blockers, ACEIs, and aldosterone antagonists can also have an important protective effect on the LVEF and can reduce the significant incidence of heart failure.

## 9. Strengths and Limitations 

The current study is the most comprehensive and has the largest sample size thus far, demonstrating the protective effect of statin on the LVEF and reducing the incidence of heart failure. There are certain limitations that are important to mention: The included studies have varying study designs, patient populations, and endpoints. Therefore, the pooled results may be influenced by this inherent heterogeneity. Furthermore, with varying follow-up periods, they may not be sufficient to capture potentially delayed effects of statins and thus may underestimate the benefits of statins. Our study includes both randomized controlled trials and observational studies. We were not able to perform subgroup analysis or regression analysis given the limited number of studies and unequal studies in each subgroup. We were not able to evaluate other variables based on echo and imaging parameters due to a limited number of studies. Our study included study-level data instead of individual patient data. Lastly, different imaging modalities such as cardiac magnetic resonance and echocardiography were used for the interpretation of the LVEF to identify the cardiotoxicity. Hence, the results should be interpreted in the context of these limitations. 

## 10. Conclusions

The results of this meta-analysis indicate that statin therapy may offer cardioprotective benefits for patients with CIC, improving left ventricular function and reducing the risk of heart failure. Further prospective randomized adequate power trials are needed to strengthen these findings and the potential beneficial effects of statin among cancer patients who are on chemotherapy. 

## Figures and Tables

**Table 1:** Baseline demographics, comorbidity, and characteristics of included studies.

**Author**		**Sample Size**	**Study Design**	**Age**	**Year**	**Cancer Type**	**DM, n**	**Dyslipidemia, n**	**HTN, n**
Nabati et al. [18]	Rosuvastatin	38	RCT	47.74 (9.70)	2018	Breast cancer	5	10	-
	Control	39		50.74 (12.440)			7	8	-
Acar et al. [12]	Atorvastatin	20	RCT	53.7 (14.2)	2011	Blood cancer			
	Control	20		52.6 (17.6)					
PREVENT (Hundley et al.) [28]	Atorvastatin	139	RCT	48.5 ± 12.5	2022	Breast cancer and lymphoma			
	Control	140		49.4 ± 11.5					
Chotenimitkhun et al. [29]	Atorvastatin	14	Observational	62 ± 2	2007–2010	Breast cancer, leukemia, and lymphoma	7	14	12
	Control	37		43 ± 2			2	2	10
Abdel-Qadir et al. (anthracyclin) [11]	Statin	859	Observational	69 (67–73)	2007–2017	Breast cancer (Antra)	317		692
	Control	1686		69 (67–72			178		915
Abdel-Qadir et al. (transtuzumab) [11]	Statin	520	Observational	71 (68–75)	2007–2017	Breast cancer (Trastu)	199		433
	Control	851		70 (68–74)			109		487
Calvillo-Argü;elles et al. [15]	Statin	43	Observational	62.0 ± 9.1	2018	Breast cancer	16	43	25
	Control	86		62.0 ± 9.0			4	9	19
Seicean et al. [30]	Statin	67	Observational	61.3 (8.9)	2005–2010	Breast cancer	17		34
	Control	561		50.3 (10.4)			15		74
SPARE-HF (Thavendiranathan et al.) [17]	Atorvastatin	54	RCT	55.2 (13.7)	2017–2022	Breast, lymphoma, leukemia, and thymoma	3	2	11
	Control	58		58.6 (13.4)			4	3	18
STOP-CA (Neilan et al.) [16]	Statin	150	RCT	50	2023	Lymphoma	0		14
	Placebo	150		49			2		20

## Data Availability

The data underlying this article are available in the article and its online Supplementary Material.

## Supplementary Materials

The following supporting information can be downloaded at https://www.mdpi.com/article/10.3390/medicina60040580/s1, Figure S1: Risk of bias in randomized controlled trials by using Cochrane Collaboration’s tool; Figure S2: Funnel plots of primary outcome: LVEF; Figure S3: Funnel plots of outcome: Heart Failure; Figure S4: Leave-one-out analysis for LVEF; Figure S5: Leave-one-out analysis for LVEDV; Table S1: Search strategy among database; Table S2: Newcastle-Ottawa scale for quality assessment and bias assessment of observational studies.
