# Supplementary material for: Association between Statin Use and Chemotherapy-Induced Cardiotoxicity: A Meta-Analysis
Source: Medicina (Kaunas). 2024 Mar 31;60(4):580. doi: 10.3390/medicina60040580 (PMC11052115; doi:10.3390/medicina60040580)
Supplement: Supplementary file 1 [file medicina-60-00580-s001.zip › medicina-2907215-supplementary.pdf]

Supplementary materials

Supplementary Table S1: Search strategy among database,

Supplementary Table S2: Newcastle-Ottawa scale for quality assessment and bias assessment of observational studies.

Supplementary Figure S1: Risk of bias in randomized controlled trials by using Cochrane Collaboration's tool.

Supplementary Figure S2 Funnel plots of primary outcome: LVEF

Supplementary Figure S3 Funnel plots of outcome: Heart Failure

Supplementary Figure S4 Leave-one-out analysis for LVEF

Supplementary Figure S5 Leave-one-out analysis for LVEDV.

Electronic database	Search strategy
Pubmed	((((((((((breast cancer[MeSH Terms]) OR (lymphoma[Other Term])) OR (cancer[Other Term])) AND (chemotherapy[Other Term])) OR (anthracyclines[Other Term])) AND (statins, hmg coa[MeSH Terms])) OR (hmg coa statins[MeSH Terms])) OR (atorvastatin[Other Term])) OR (simvastatin[Other Term])) AND (heart failure[MeSH Terms])) OR (cardiomyopathies[MeSH Terms])

Supplementary Table S1: Search strategy

Study	Selection	Comparability	Outcome	To

**Author, year**	Representatives of exposed group	Selection of non-exposed cohort	Ascertainment of exposure	Outcome of interest		Outcome assessment	Adequacy of follow up duration	Adequacy of follow up of cohort	
**Qadir et al**	1	1	1	1	2	1	0	0	7
**Arguell et al.**	1	1	1	1	2	1	1	1	9
**Seicean et al.**	1	1	1	1	2	1	0	0	7
**Chotenimitkhun et al.**	1	1	1	1	1	1	1	1	8

Supplementary Table S2. Newcastle-Ottawa scale for quality assessment and bias assessment of observational studies.

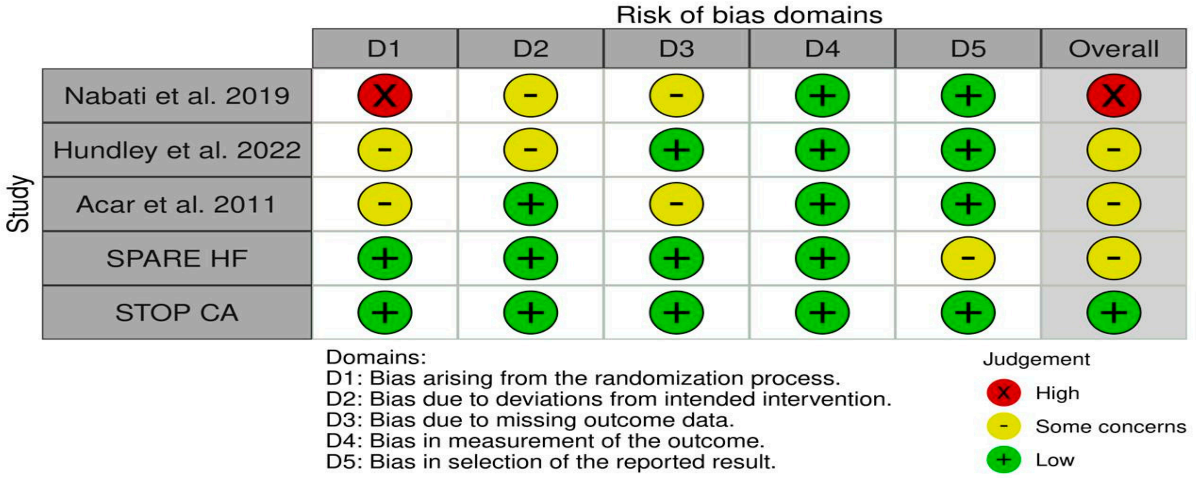

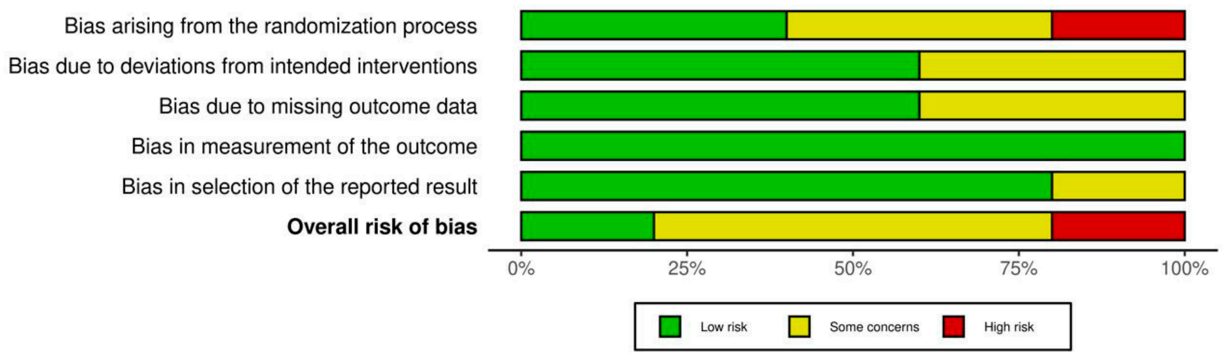

Supplementary Figure S1: Risk of bias in randomized controlled trials by using Cochrane Collaboration's tool.

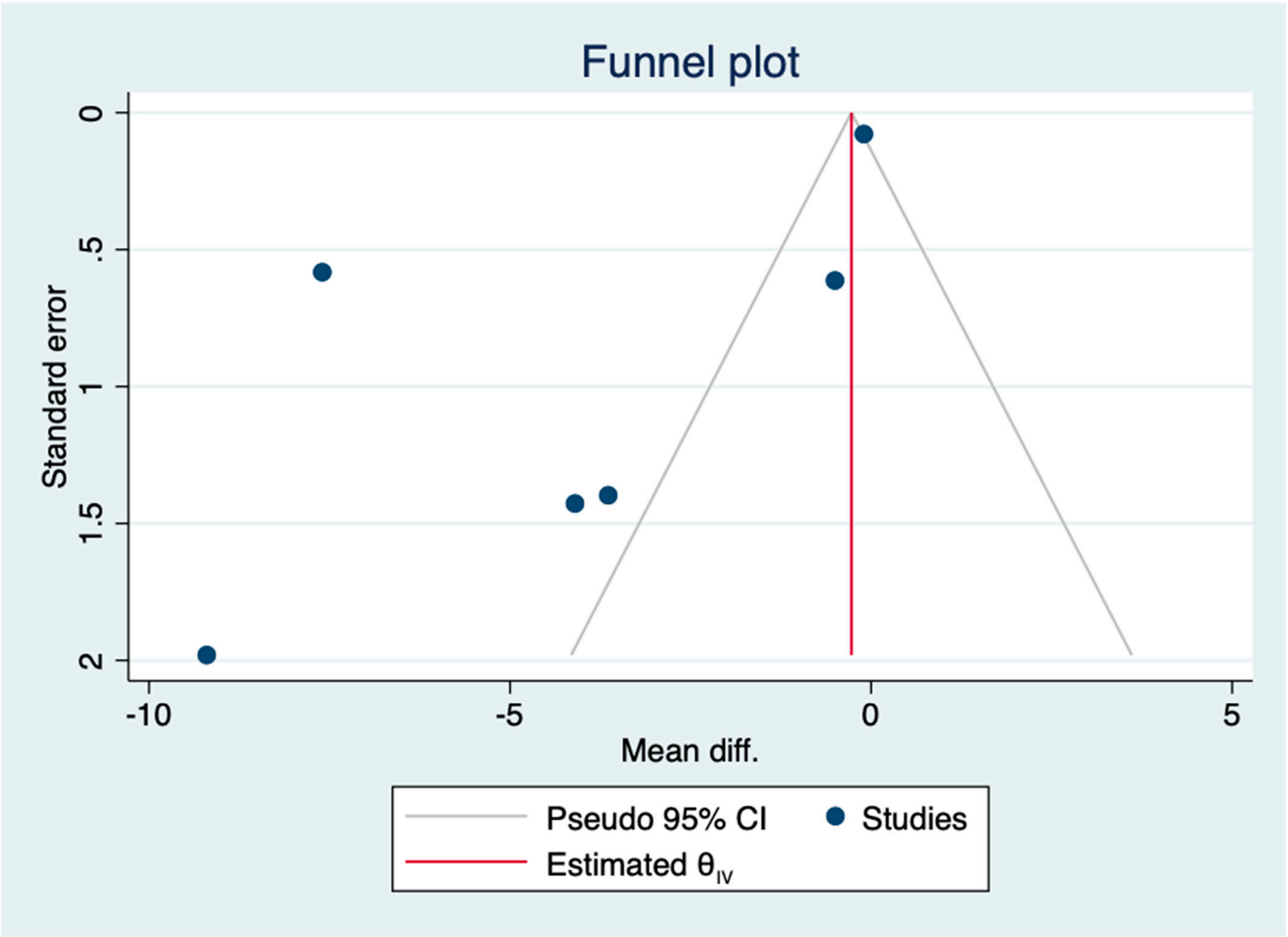

Supplementary Figure S2 Funnel plots of primary outcome: LVEF

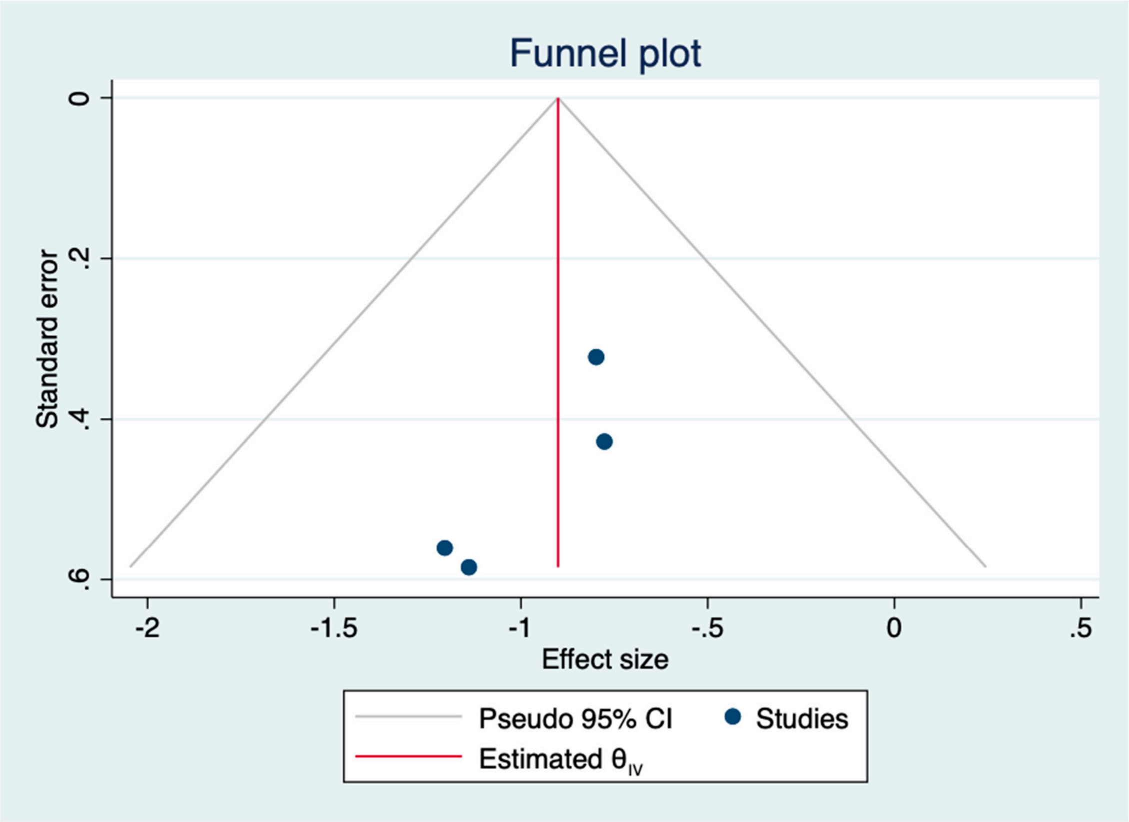

Supplementary Figure S3 Funnel plots of outcome: Heart Failure

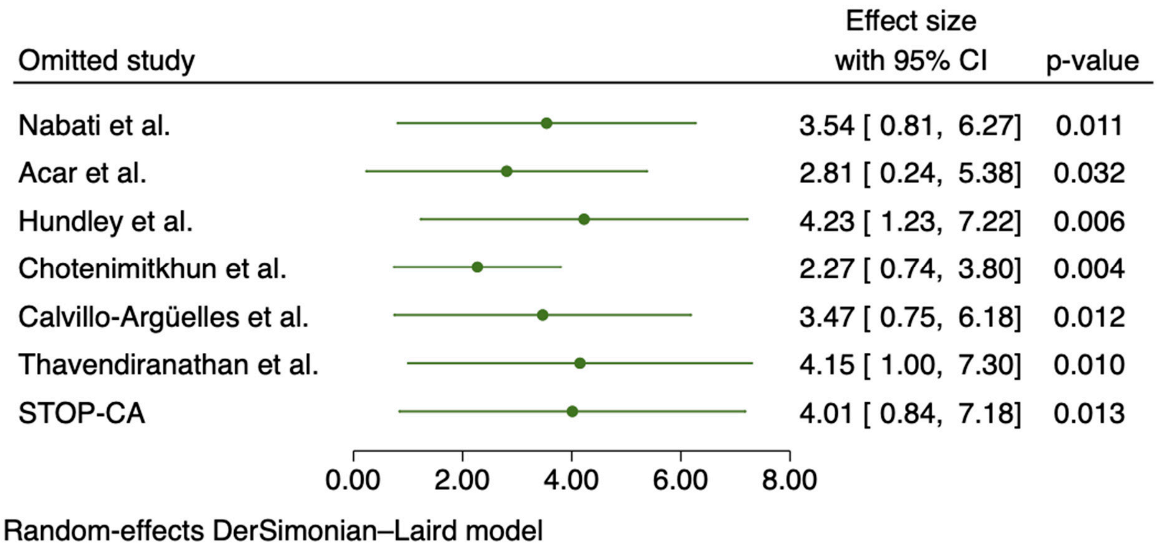

Supplementary Figure S4 Leave-one-out analysis for LVEF

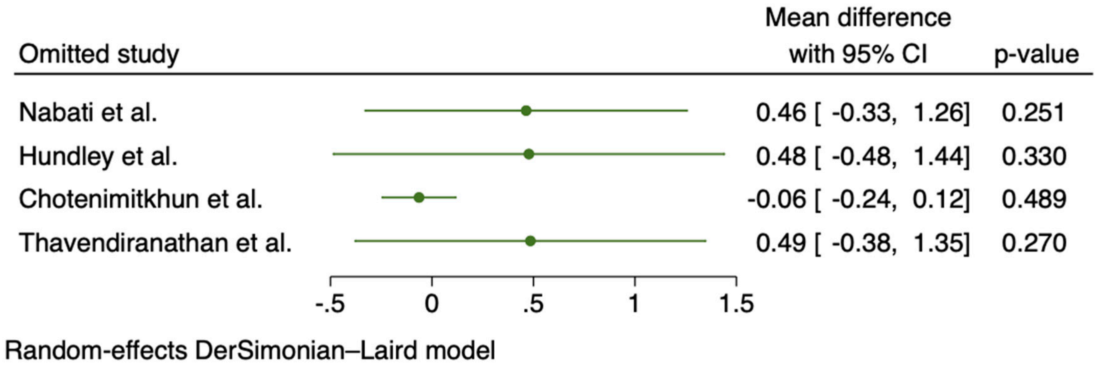

Supplementary Figure S5 Leave-one-out analysis for LVEDV.
